# Metagenomic and Metabolomic Insights into Volatile Flavor Changes and Microbial Community Shifts in *Physalis pubescens* L. Fermentation by *Lactiplantibacillus plantarum*

**DOI:** 10.3390/molecules31132377

**Published:** 2026-07-06

**Authors:** Song Yan, Jialei Li, Kaixin Chen, Chuanying Ren, Shan Zhang, Qing Chen, Yang Gao, Bin Liu

**Affiliations:** 1 Food Processing Research Institute, Heilongjiang Academy of Agricultural Sciences, Harbin 150086, China; 2 College of Pharmacy, Harbin University of Commerce, Harbin 150028, China

**Keywords:** *Physalis pubescens* L., *Lactiplantibacillus plantarum*, metagenomics, metabolomics, volatile flavor compounds, fermentation

## Abstract

*Physalis pubescens* L. is a seasonal fruit with high nutritional value but a short shelf life that limits its processing and utilization. This study integrated metagenomics and metabolomics to investigate the comparative effects of *Lactiplantibacillus plantarum* fermentation on volatile flavor metabolites and microbial community composition of *P. pubescens* by comparing initial (0 h) and post-fermentation (24 h) states. After 24 h of fermentation, 1316 volatile compounds were putatively identified by GC-MS, with 592 metabolites significantly changed and 501 upregulated and 91 downregulated. Key flavor compounds that impart citrus, floral, fruity, and rose notes including D-limonene, geraniol, D-carvone, and phenylethyl alcohol were markedly increased. Metagenomic analysis revealed that *L. plantarum* rapidly dominated the microbial community (relative abundance surged from <0.05% to ~72%) while effectively suppressing potential spoilage bacteria such as *Escherichia coli*. Functional gene annotation demonstrated significant enrichment of amino acid, carbohydrate, and fatty acid metabolism pathways, with key enzyme genes (L-lactate dehydrogenase, pyruvate oxidase, acetyl-CoA carboxylase) predominantly assigned to *L. plantarum*, suggesting their potential contribution to the generation of organic acids, ethanol, and esters. Spearman correlation analysis indicated that *Lactobacillaceae genera* were significantly positively correlated with terpenoids, phenols, alcohols, and aldehydes. This study provides the first metagenomics-metabolomics insight into the microbial and molecular mechanisms associated with flavor formation in LAB-fermented *P. pubescens*, offering a theoretical foundation for developing stable and controllable fermented fruit products.

## 1. Introduction

*Physalis pubescens* L. is an annual herbaceous plant of the genus Physalis in the family Solanaceae, and its ripe fruit appears bright golden yellow [1]. Originally native to the American continent, it is now widely distributed in Chinese provinces such as Jilin and Heilongjiang, where it has become a specialty agricultural product of Northeast China [2]. The fruit of *P. pubescens* has high nutritional and edible value [3]. *P. pubescens* is not only convenient to eat and unique in flavor, but also rich in trace elements, various vitamins, natural pigments, and eighteen kinds of amino acids [4]. With in-depth research on its chemical composition and biological activities, it has been found that this plant contains a variety of active components and exhibits significant pharmacological effects, including antioxidant, anti-inflammatory, antibacterial, and antitumor activities [5,6]. In previous studies, various phytochemicals have been isolated and identified from the fruits of *P. pubescens*, including esters [7], terpenoids [8], as well as cinnamoyl and hydroxycinnamoyl derivatives [4]. However, *P. pubescens* is a seasonal fruit characterized by a limited storage window and a short shelf life. Lactic acid bacteria (LAB) are the most widely used group of bacteria in the food industry, with their prominent roles reflected in the preservation and flavor development of fruit and vegetable juices [9,10]. At the same time, LAB also play a key role in various physiological functions, including the regulation of intestinal microecology-related diseases [11,12]. Among them, *Lactiplantibacillus plantarum* possesses an outstanding ability to ferment a wide range of carbohydrates and adapt to diverse growth environments. This strong adaptability makes it an important player in the fermentation of commercial plant-based foods, helping to improve sensory quality, extend shelf life, and ensure safety [13]. Therefore, *L. plantarum* shows promising application prospects in the field of fruit and vegetable juice fermentation. However, little is known about its role in flavor formation and volatile distribution after fermentation of *P. pubescens*, and the underlying molecular mechanisms remain unexplored.

The fermentation process of traditional foods relies on the synergistic action of diverse microbial communities, which secrete hydrolytic enzymes that break down carbohydrates and proteins. Through a series of complex biochemical reactions, these processes ultimately generate the desired flavor compounds [14]. With the rapid advancement of molecular biology, metagenomics has been employed to analyze the dynamics of microbial communities during fermentation, as exemplified in studies on fish sauce [15], potherb mustard (*Brassica juncea var. multiceps*) [16], and huangjiu (Chinese rice wine) [17]. The introduction of metagenomic technologies has enabled us to resolve the complex microbial community structures in fermented foods, further advancing our understanding of the metabolic and functional potential of microbial communities and how they influence the quality of the final product. To date, numerous studies on fermented foods have integrated correlation analysis and validation approaches to deeply elucidate the specific roles of microorganisms in aroma formation, as demonstrated in research on *Capsicum annuum* L. [18], JIUYao [19], and broad bean paste [20].

Despite these advances, several critical knowledge gaps remain. First, while metagenomics has been applied to various fermented foods, no study has yet employed this approach to investigate the mechanism of flavor formation in fermented *P. pubescens*. Second, although the volatile compounds of *P. pubescens* have been characterized in other contexts, the community composition of flavor metabolites after LAB fermentation and their correlation with microbial community succession have not been systematically studied. Third, the integration of metagenomics and metabolomics, which has proven powerful for elucidating microbe-metabolite relationships in other fermented foods, has not been applied to *P. pubescens* fermentation. Addressing these gaps is important not only for understanding the fundamental biology of *L. plantarum* in fruit matrices but also for developing stable, controllable, and high-quality LAB-fermented fruit products.

Therefore, this study aims to integrate metagenomic and metabolomic approaches to characterize the diversity patterns of microbial populations and elucidate their associations with volatile compounds after *L. plantarum* fermentation of *P. pubescens*. Specifically, we intend to: (i) systematically compare the changes in flavor metabolites between initial and post-fermentation states; (ii) resolve the structure and function of the corresponding microbial communities, identifying the dominant bacterial species; (iii) identify the key enzyme genes and metabolic pathways responsible for flavor compound generation; and (iv) establish the correspondence between core microorganisms and major flavor compounds through correlation analysis. By jointly analyzing metagenomic data and volatile metabolites, the intrinsic links between the microbial community and its volatile metabolic profile can be effectively revealed. To the best of our knowledge, this is the first study to provide a metagenomics-metabolomics elucidation of the microbiological and molecular mechanisms by which *L. plantarum* drives flavor formation in *P. pubescens*, offering a theoretical foundation for developing stable and controllable LAB-fermented fruit and vegetable products.

## 2. Results and Discussion

### 2.1. Physicochemical Property Changes in Physalis pubescens L. After 24 h Fermentation

**Figure 1:**
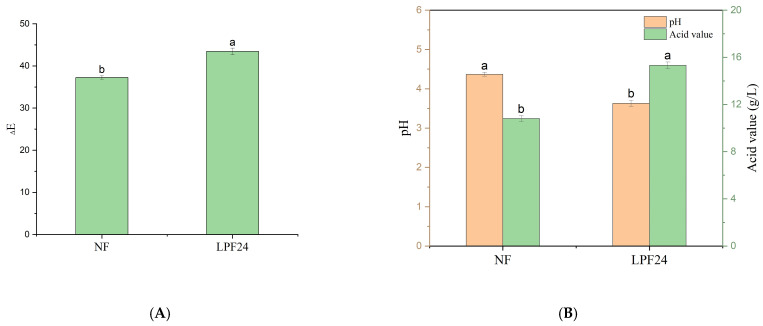
Physicochemical properties of *Physalis pubescens* L. before and after fermentation. (**A**) Color difference (ΔE) values of the non-fermented (NF) and *L. plantarum*-fermented for 24 h (LPF24) groups. (**B**) pH values and total acid (TA) content of the NF and LPF24 groups. Data are presented as mean ± standard deviation (*n* = 3). Different letters indicate significant differences between groups (*p* < 0.05).

The flavor quality of fermented vegetables is closely linked to the composition and activity of their microbial community, which is in turn significantly influenced by environmental factors such as pH, titratable acidity (TA), and salinity [21]. The color difference, pH, and total acid (TA) content of *P. pubescens* before and after inoculated fermentation were measured (Figure 1). Color is an important quality indicator of fermented foods. Compared with the control group (Uninoculated, non-fermented group, abbreviated as NF group), the ∆E value of the experimental group (*Lactiplantibacillus plantarum*-inoculated, fermented for 24 h group, abbreviated as LPF24 group) increased from 37.26 to 43.44 (*p* < 0.05), indicating a significant enhancement in fruit color after fermentation, which appeared as a brighter golden-yellow hue. This change may be attributed to pigment dissolution or structural modification mediated by organic acids produced by *Lactiplantibacillus plantarum* metabolism, or it may originate from the lower pH environment of the fermented juice, which enhances the color expression of anthocyanins and increases their color difference parameters [22]. The pH decreased significantly from 4.37 in the NF group to 3.63 in the LPF24 group (*p* < 0.05), while the total acid content increased from 10.81 g/L to 15.32 g/L (*p* < 0.05). The increase in acidity is a typical characteristic of lactic acid bacterial fermentation. *L. plantarum* converts sugars into lactic acid via the homolactic fermentation pathway, potentially accompanied by acetic acid production, thereby rapidly accumulating organic acids and lowering the system pH [21]. This acidified environment not only imparts a refreshing sour taste to the product but also helps inhibit spoilage and pathogenic microorganisms, which may contribute to extending the product’s shelf life.

### 2.2. Volatile Flavor Metabolite Changes in Physalis pubescens L. After 24 h Fermentation

**Figure 2:**
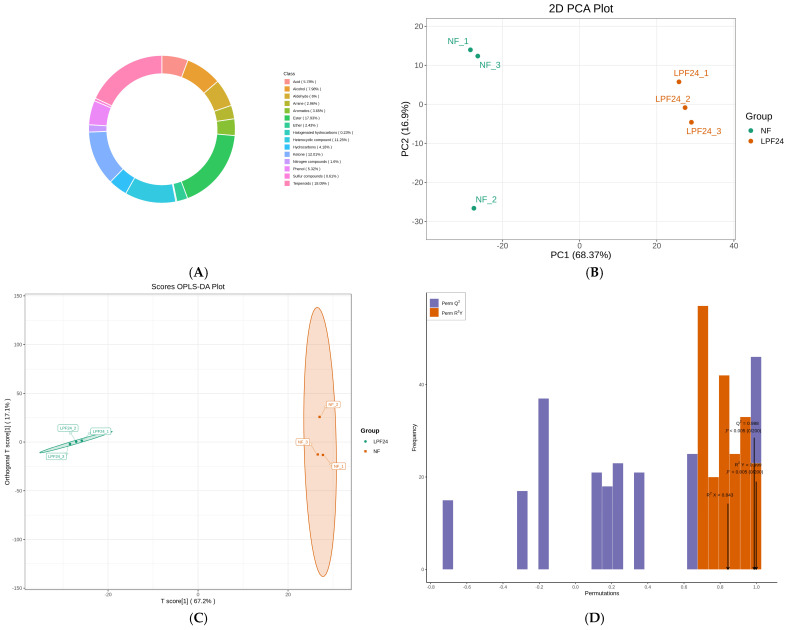

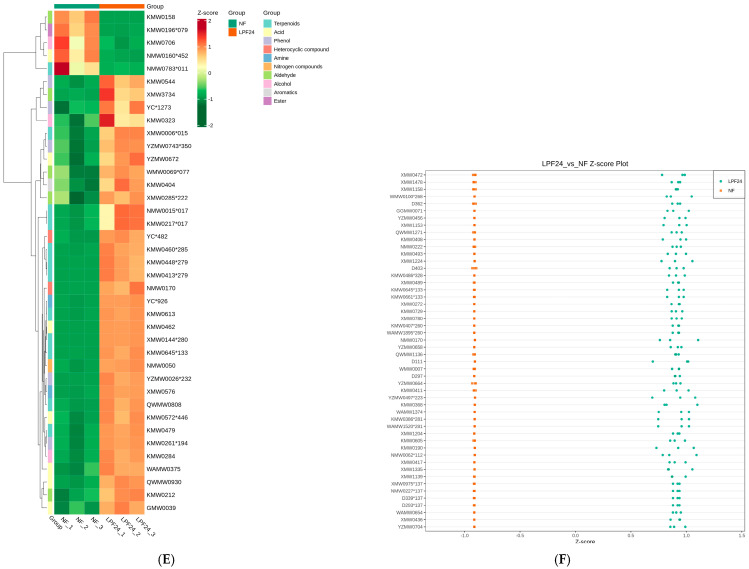
Volatile flavor metabolite profiles of *Physalis pubescens* L. before and after 24 h fermentation. (**A**) Classification and counts of volatile compounds detected by GC-MS. (**B**) Principal component analysis (PCA) score plot of all samples (NF and LPF24 groups). (**C**) Orthogonal partial least squares discriminant analysis (OPLS-DA) score plot showing separation between NF and LPF24 groups. (**D**) Permutation test (200 permutations) for OPLS-DA model validation; all permuted R^2^ and Q^2^ values were lower than the original values, confirming model validity. (**E**) Heatmap of differential metabolites (VIP > 1, FC ≥ 2 or ≤0.5); red indicates high relative abundance, green indicates low relative abundance. (**F**) Z-score plot of the top 50 differential metabolites ranked by VIP values.

Flavor is a key attribute that defines the quality and consumer acceptance of fermented foods [23]. Using GC-MS technology, the volatile compounds of NF group and LPF24 group of *P. pubescens* were analyzed. A total of 1316 volatile compounds were detected, including 238 terpenoids, 236 esters, 159 ketones, 148 heterocyclic compounds, 105 alcohols, 79 aldehydes, 76 acids, 70 phenols, 48 aromatic hydrocarbons, 32 ethers, and other compounds (Figure 2A). The results showed that fermentation significantly affected the contents of most volatile compounds, with the most pronounced effects observed among terpenoids, phenols, acids, ketones, and esters. The types and contents of volatile flavor compounds in fruit and vegetable fermentation such as esters, acids, and terpenoids depend on the substrate composition, microorganisms (especially LAB), and fermentation conditions [24,25]. From the NF group to the LPF24 group, these substances all showed a sharp increasing trend, with terpenoids and phenols accumulating the most after fermentation. Terpenoids are important components of plant-derived aromas, and their increase may enhance the fresh, citrus, and floral notes of the product [9]. The changes in phenolic compounds may originate from the metabolic activity of *L. plantarum* promoting the conversion or release of phenolic precursors [26]. Phenolics are generally associated with antioxidant, antimicrobial, and flavor-enhancing properties; their substantial increase suggests that the fermentation process may enhance the functionality and flavor complexity of *P. pubescens* [27]. This obvious trend highlights the beneficial effects of *L. plantarum* fermentation on *P. pubescens*, ultimately leading to a transformative enhancement of its sensory characteristics. The accumulation of acids is likely related to the acid-producing metabolism of LAB and has a significant impact on product pH and flavor stability. Ketones often impart fruity and creamy notes; their increase may enhance the flavor complexity of the fermented product. Esters typically have floral and fruity aromas; their accumulation may be related to the esterase activity of lactic acid bacteria, contributing to flavor roundness and harmony.

Principal component analysis (PCA) was performed on the differential metabolites of *P. pubescens* before and after fermentation (Figure 2B). The PCA results were similar to those from orthogonal partial least squares discriminant analysis (OPLS-DA), with clear separation between samples before and after fermentation (Figure 2C). Permutation tests indicated that the model had good fitting ability and predictive power (Figure 2D). In addition, a total of 592 metabolites were putatively identified as differential flavor substances (VIP > 1, fold change ≥ 2 or ≤0.5), of which 501 were upregulated and 91 were downregulated. Notably, these differential compounds showed distinct distributions before and after fermentation. Heatmap analysis was performed on volatile compounds involved in key metabolic pathways before and after fermentation (Figure 2E). Beyond demonstrating statistical separation, the multivariate analyses provide mechanistically meaningful insights into the metabolic reprogramming induced by *L. plantarum* fermentation. The PCA score plot (Figure 2B) revealed clear segregation between NF and LPF24 samples along the first principal component (PC1), indicating that fermentation is the dominant source of metabolic variation in the dataset. The tight clustering within each group demonstrates high reproducibility among biological replicates and confirms that the fermentation process yielded consistent metabolic outcomes. Since PCA is an unsupervised method that does not incorporate any class information, this clear separation provides strong evidence that the metabolic differences between groups are intrinsic and robust, rather than artifacts of data processing. The OPLS-DA model (Figure 2C) further corroborated this separation with enhanced resolution. As a supervised method, OPLS-DA incorporates class labels during modeling and separates predictive variation (related to the class difference) from orthogonal variation (unrelated to the class difference). The distinct clustering observed in the score plot indicates that the metabolic differences between NF and LPF24 groups are systematic and not driven by random noise or individual variability. The permutation test (Figure 2D, 200 permutations) served as a rigorous validation against overfitting in supervised multivariate analysis. The hierarchical clustering heatmap (Figure 2E) revealed two fundamental biological patterns. First, row-wise clustering grouped metabolites with similar accumulation patterns across samples, indicating that certain classes of compound (particularly terpenoids, esters, and phenols) are co-regulated during fermentation. This co-regulation likely reflects shared biosynthetic origins. Second, column-wise clustering showed that all LPF24 samples clustered tightly together and separately from all NF samples, indicating that fermentation exerts a uniform and reproducible effect on the metabolome.

Specifically, terpenoids were the largest class of volatile compounds. Among them, compounds such as D-limonene, nerol, (−)-carvone, safranal, alpha.-farnesene, Citronellol, (−)-caryyl acetate, trans-.beta.-ocimene, (+)-2-bornanone, D-carvone, etc., had low abundances in NF samples but generally showed significant upregulation after fermentation. These volatile organic compounds contribute green, floral, fruity, and herbal aromas. D-limonene and β-damascone are both important flavor terpenoids; studies have shown that their contents increase after juice fermentation [28,29,30]. Some other terpenoids, such as Geraniol, also increased significantly after fermentation. Research has indicated that among juices fermented with *L. plantarum*, *Lactobacillus casei*, and *Lactobacillus rhamnosus*, the highest Geraniol concentration was observed after *L. plantarum* fermentation, while the lowest was in unfermented juice [31,32]. Ester compounds are known for their fruity and floral aromas. For example, β-phenylethyl acetate, Benzenepropanoic acid-ethyl ester, and p-menth-8-en-3-ol-acetate increased in content with fermentation. This accumulation is consistent with enhanced esterification activity due to microbial metabolism, contributing to the sweetness and fruitiness of fermented *P. pubescens* [24,33]. However, some esters, such as (Z)-3-hexen-1-ol-acetate decreased in content after fermentation. In addition, the contents of alcohols (e.g., benzyl alcohol and phenylethyl alcoho), ketones (e.g., 3-octen-2-one and 1-(2-methylphenyl)ethanone), and aldehydes (e.g., 3-phenyl-2-propenal and Benzeneacetaldehyde) all increased after fermentation. These flavor compounds add fruity, rose, green, and mushroom-like odors to the fermented *P. pubescens*. Studies on mango juice fermented with 40 different *L. plantarum* strains demonstrated that fermentation significantly enhanced the complexity and intensity of aroma compounds, especially terpenes, esters and alcohols, with esters playing a key role in enhancing fruity and floral aromas [34]. In carrot pulp fermented by *L. plantarum* NCU116, increased levels of organic acids, improved amino acid conversion, and higher contents of ketones, alcohols, and esters were observed, imparting fruity and floral flavors [35]. These collective observations across diverse fruit substrates are consistent with the view that the flavor-enhancing effects of *L. plantarum* fermentation, particularly the accumulation of terpenoids, esters, and alcohols, represent a general and robust phenomenon, rather than being specific to *P. pubescens*. The relative abundances of differential metabolites in different samples were subjected to Z-score standardization (i.e., unit variance scaling, UV), and the distribution of the top 50 differential metabolites with the highest VIP values among groups was visualized (Figure 2F).

### 2.3. Microbial Community Structures and Functions

**Figure 3:**
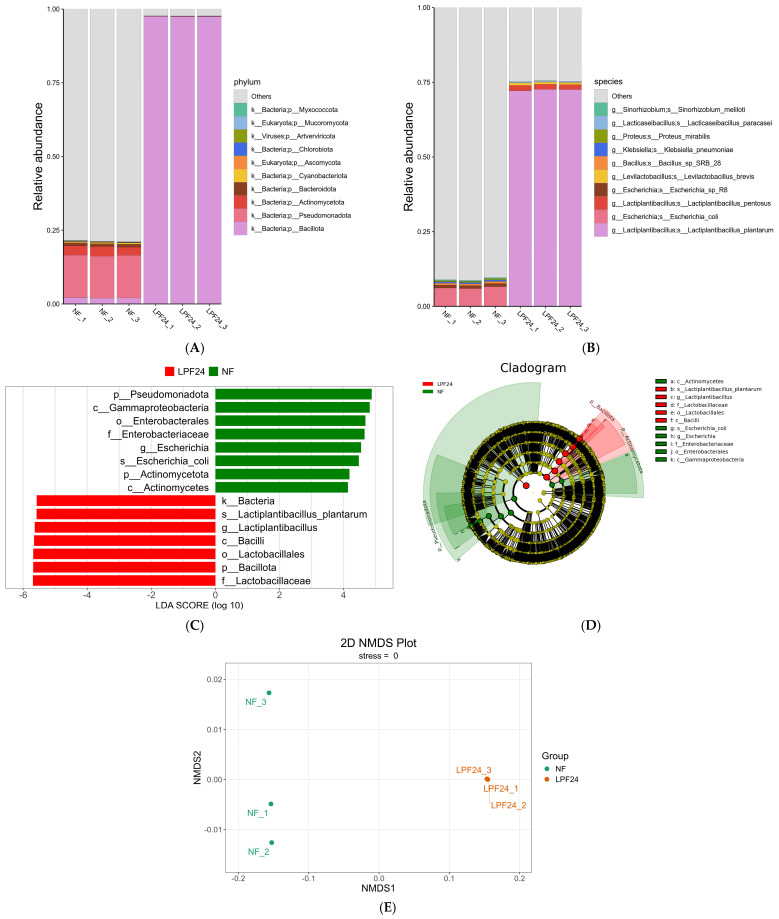
Microbial community shifts in *Physalis pubescens* L. after 24 h fermentation with Lactiplantibacillus plantarum. (**A**) Relative abundance of microbial community composition at the phylum level before (NF) and after (LPF24) fermentation. (**B**) Relative abundance at the species level before and after fermentation. (**C**) Linear discriminant analysis effect size (LEfSe) distribution bar plot showing biomarkers distinguishing NF and LPF24 groups. (**D**) LEfSe cladogram displaying taxonomic differences between groups. (**E**) Non-metric multidimensional scaling (NMDS) analysis based on Bray–Curtis distance of functional abundance, showing clustering of LPF24 samples separate from NF samples.

The metagenomic sequences were compared against the NCBI nr database to identify the putative dominant taxa in each experimental group. In the uninoculated, non-fermented group (NF group), the others category accounted for approximately 78%, indicating that no single species dominated the original community and the community structure was highly dispersed. The main components were Bacteria, Eukaryota, and Viruses. At the phylum level, the abundances from highest to lowest included *Pseudomonadota* (14.39%), *Actinomycetota* (3.17%), *Bacillota* (2.16%), *Bacteroidota* (1.01%), *Cyanobacteriota* (0.41%), Ascomycota (0.10%), *Chlorobiota* (0.10%), *Artverviricota* (0.08%), *Mucoromycota* (0.05%), and *Myxococcota* (0.04%). The sequencing results of the NF group actually represent the total DNA of all microorganisms carried by the raw materials themselves and introduced during production and processing. These diverse microbial communities likely originate from the raw materials [36] and are mainly involved in processes such as cellulose degradation, pectin decomposition, absorption and cleavage of sugar residues, and amino acid metabolism [16,37]. The fermentation process led to a fundamental remodeling of the microbial community. After inoculation with *L. plantarum*, the community in LPF24 group rapidly shifted to dominance by *Bacillota* (from 2.16% to 97%), while other phyla were suppressed, marking a successful transition of the fermentation system from ‘environmental’ to ‘lactic acid fermentation type’ (Figure 3A,C). The LEfSe cladogram also indicated that microbial changes began at the phylum level following inoculation (Figure 3C,D).

At the species level, *L. plantarum* grew rapidly and became absolutely dominant after inoculation. Within 24 h post-inoculation, the relative abundance of *L. plantarum* surged from less than 0.05% in the NF group to approximately 72%, successfully achieving mono-dominant colonization (Figure 3B). Through metabolomics analysis, it was found that *L. plantarum* B7 grew rapidly during the fermentation process, and the bacterial count stabilized after 24 h [38]. NMDS analysis further revealed that the community structures of all inoculated treatment samples were highly clustered and significantly separated from the NF control group, demonstrating the high community convergence resulting from mono-dominant colonization (Figure 3E). This indicates that under the established fermentation conditions, this strain exhibits strong competitiveness and adaptability to the *P. pubescens* matrix, enabling it to dominate the fermentation system within a very short period. Of particular note, the potential contaminating bacteria *Escherichia coli* and *Escherichia* sp. R8, which were relatively abundant in the NF group, dramatically decreased to negligible levels (relative abundance < 0.0001%) after inoculation. This ‘clearance effect’ suggests that *L. plantarum*, through rapid acid production and niche competition, can efficiently inhibit potentially harmful microorganisms present in the environment, thereby significantly enhancing the safety of the fermentation system [39]. It should be noted that the detection of potential contaminants such as *Escherichia coli* in the pasteurized NF group does not indicate their survival as viable cells, as the pasteurization condition (85 °C, 10 min) is sufficient to inactivate vegetative pathogens. Instead, their presence in the metagenomic dataset is attributable to the persistence of relic DNA fragments from dead cells, which are captured by high-throughput sequencing. The drastic reduction in these signals after 24 h of fermentation further confirms that the robust acidification and niche dominance of the starter culture effectively suppressed any remaining DNA traces or potential spore germination, contributing to the safety and stability of the fermented product. Although *L. plantarum* was absolutely dominant, a weak but significant proliferation of two other LAB was still observed: *Levilactobacillus brevis* and *Lactiplantibacillus pentosus*. They were nearly undetectable in the NF group but reached abundances of approximately 0.78% and 1.7%, respectively, after 24 h of fermentation. This phenomenon suggests that within a fermentation system dominated by *L. plantarum*, there may exist a simplified but non-single microbial community. These accompanying strains share similar ecological niches (acid tolerance, lactic acid production) with the dominant bacterium, but their proliferation ability is relatively weak, so they only coexist at low abundances. Their presence may potentially contribute to the complexity and layering of fermentation flavors, warranting further investigation through metabolomics.

From a macro perspective of community structure, the proportion of ‘other species’ in the NF group exceeded 92%, reflecting the highly dispersed and unpredictable nature of the residual DNA from dead or inactivated microorganisms remaining after pasteurization of the raw materials. However, after 24 h of inoculated fermentation, the proportion of ‘other species’ dropped sharply to approximately 24%, indicating that massive inoculation of *L. plantarum* successfully outcompeted the residual dead-cell DNA signals and occupied the fermentation ecosystem. This community structure shift from ‘highly dispersed, low predictability’ to ‘low dispersion, high controllability’ is a direct manifestation of the advantages of the inoculated fermentation process. From a microbial ecology perspective, this study provides a solid theoretical basis for developing a safe, stable, and controllable lactic acid fermentation process for *P. pubescens*.

To understand the substrate basis underlying the rapid dominance of *L. plantarum* in the *P. pubescens* fermentation system, we surveyed the chemical composition of this fruit from the literature. *P. pubescens* fruits contain approximately 10.85% carbohydrates [40], with the sugars being predominantly fructose and glucose readily fermentable monosaccharides that *L. plantarum* can efficiently utilize via the glycolytic pathway. In addition, the fruit contains a novel polysaccharide (PPL-1) composed of rhamnose, arabinose, fructose, mannose, and glucose [6]. These polysaccharides may serve as prebiotic substrates that further support the proliferation of lactic acid bacteria. The crude protein content of the fruit is 2.08% [40], and the juice is a rich source of essential amino acids, including isoleucine (4.2 g/100 g protein), valine (3.9 g/100 g protein), and tryptophan (3.9 g/100 g protein) [41]. In total, the fruit contains 18 kinds of amino acids, with aspartic acid and leucine being the most abundant, followed by arginine, glycine, and valine. This abundant and diverse pool of carbohydrates (simple sugars, starch, and polysaccharides) and proteins/amino acids provides the ideal nutritional foundation for *L. plantarum*, a species renowned for its ability to ferment a wide range of carbohydrates and adapt to diverse environments to rapidly establish dominance.

**Figure 4:**
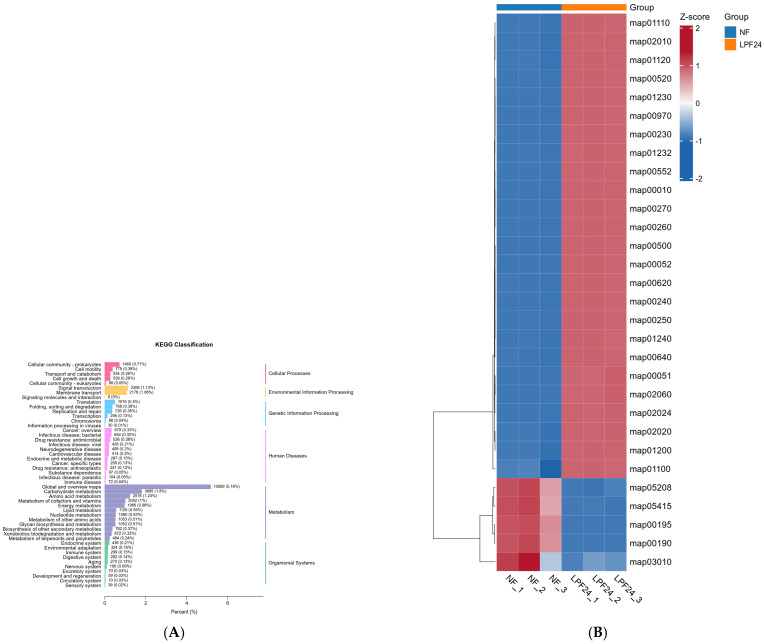
KEGG functional classification of the *Physalis pubescens* L. microbial community before and after inoculated fermentation. (**A**) Statistical chart of the number of annotated genes from each database. (**B**) Heatmap of functional predictions for the 30 most abundant level-3 KEGG orthologs in the community before and after inoculated fermentation. Color intensity reflects relative abundance of each functional pathway.

To gain a deeper understanding of the functional pathways associated with the identified genes, the genes were mapped to the KEGG pathway database. In the annotated dataset, a total of 444 metabolic subsystems were putatively identified at KEGG classification level 3. These genes were associated with amino acid metabolism, carbohydrate metabolism, and the global and overview maps of KEGG gene function level 2. Among the level-2 KEGG pathways (Figure 4A), amino acid metabolism and carbohydrate metabolism exhibited the highest relative abundances. The high abundance of genes involved in amino acid and carbohydrate metabolism indicates that the material basis for flavor formation is primarily proteins and starch.

Based on the functional annotation and abundance information of all samples across various databases, a heatmap was generated using the top 30 functions ranked by abundance at KEGG Level 3, along with their abundances in each sample, and clustering was performed from the perspective of functional differences. Among these pathways, the following were relatively abundant during the fermentation of *P. pubescens*: Biosynthesis of amino acids (map01230), Starch and sucrose metabolism (map00500), Fructose and mannose metabolism (map00051), Pyruvate metabolism (map00620), Propanoate metabolism (map00640), Glycolysis/Gluconeogenesis (map00010), Cysteine and methionine metabolism (map00270), Glycine, serine and threonine metabolism (map00260), and Alanine, aspartate and glutamate metabolism (map00250) (Figure 4B). These abundant pathways are of particular interest to this study, as they are plausibly linked to the production of flavor compounds in fermented *P. pubescens*.

### 2.4. Analysis of Bacterial Genera Associated with Flavor Formation

**Figure 5:**
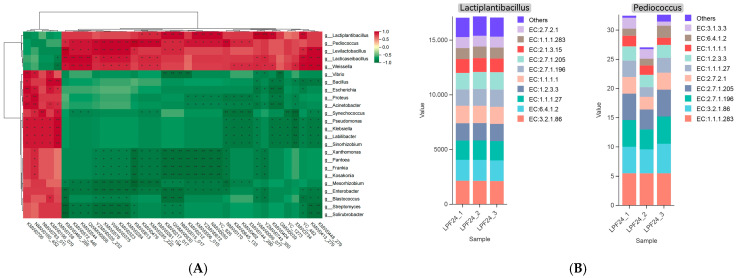
Core microbes and potential metabolic network for key flavor formation. (**A**) Correlation between the differential characteristic flavor compounds and microbiota (relative abundance > 0.1%) based on Spearman correlation efficient (|r| > 0.8, *p* < 0.05). Red indicates positive correlation, blue indicates negative correlation. (**B**) Relative abundance of enzyme-encoding genes corresponding to the dominant genera (*Lactiplantibacillus* and *Pediococcus*).

The five genera most closely associated with flavor formation are Pediococcus, *Lactiplantibacillus*, *Lacticaseibacillus*, *Weissella*, and *Levilactobacillus*. Correlation analysis was used to identify microbial taxa that might influence quantitative traits of metabolites after *P. pubescens* fermentation. Spearman’s rank correlation analysis between microbial taxonomic abundances and volatile substances revealed that volatile compounds such as terpenoids, phenols, alcohols, and aldehydes were significantly positively correlated with the abundances of *Pediococcus*, *Lactiplantibacillus*, *Levilactobacillus*, *Lacticaseibacillus*, and *Weissella* (Figure 5A; *p* < 0.01).

Furthermore, the contribution of dominant genera to the abundance of enzyme-encoding genes was associated with the metabolism of major flavor components in fermented *P. pubescens*, consistent with the abundance data, *Lactiplantibacillus* was the primary host of these enzyme genes, followed by Pediococcus (Figure 5B). The main genes and enzymes involved in metabolic pathways related to flavor formation in the genome of inoculated fermented *P. pubescens* are shown in Table 1. These observations are consistent with the hypothesis that *Lactiplantibacillus*-related genera may play an important role in shaping the unique characteristics of fermented *P. pubescens*. Based on the results of metagenomics and metabolomics analyses, we further explored the relevant data, focusing on the reaction processes involved in the metabolism of major flavor components. Regarding carbohydrate metabolism, genes encoding enzymes for the utilization of carbohydrates such as starch, sucrose, and fructose were mainly assigned to the genus *Lactiplantibacillus*. Glucose and fructose are converted to pyruvate via the glycolytic pathway. Pyruvate, as a key intermediate in carbohydrate catabolism, is a key intermediate that may contribute to the synthesis of organic acids, ethanol, and ester compounds, and also serves as an important flavor precursor [17,42]. During the fermentation of *P. pubescens*, microorganisms can metabolize carbohydrates to form organic acids, such as lactic acid and acetic acid. These abundant organic acids are important components that impart unique acidity, mouthfeel, and flavor to the fermented product [36]. After fermentation, the enrichment of key enzymes in the glycolysis/gluconeogenesis and pyruvate metabolism pathways signifies a significant enhancement in the energy metabolism of the microbial community or the plant tissue itself. The genomes of *Lactiplantibacillus* and *Pediococcus* contain genes necessary for pyruvate metabolism, including those encoding L-lactate dehydrogenase (EC 1.1.1.27) and alcohol dehydrogenase (EC 1.1.1.1), which are typical molecular markers of lactic acid fermentation and alcoholic fermentation. This is consistent with the accumulation of lactic acid and ethanol in the fermented *P. pubescens* broth, both of which are core substances affecting product acidity, flavor, and preservability. The pathway for acetic acid production in fermented *P. pubescens*. Proceeds via pyruvate oxidase (EC 1.2.3.3) and acetate kinase (EC 2.7.2.1), and these two enzymes are encoded by the genomes of *Pediococcus* and *Lactiplantibacillus*. The possible coexistence and interconversion of lactic acid, ethanol, and acetic acid may constitute to the biochemical basis for the complexity of fermentation flavors. As fermentation proceeds, some microorganisms recycle environmental acetic acid via acetate kinase to generate acetyl-CoA. Then, acetyl-CoA carboxylase (EC 6.4.1.2) is activated, acetyl-CoA carboxylase is a key node connecting carbohydrate metabolism and lipid synthesis, and its presence suggests that part of the carbon flux may be directed toward the fatty acid synthesis pathway, which may be related to the aroma precursors produced during fermentation [43]. The metabolic shift in *L. plantarum* under aerobic conditions clearly indicates that pyruvate oxidase and acetate kinase jointly participate in the metabolic pathway from lactate to acetate, and drive the generation of adenosine triphosphate (ATP) through the production of acetyl phosphate (Acetyl-Pi) [44]. Therefore, *L. plantarum* accelerates organic acid production by rapidly establishing dominance in the early stage of fermentation, thereby inhibiting harmful microorganisms and achieving the effect of acidification and antibacterial action [45]. In the starch and sucrose metabolism pathway, the enrichment of 6-phospho-beta-glucosidase (EC 3.2.1.86), oligo-1,6-glucosidase (EC 3.2.1.10), and a series of protein-Npi-phosphohistidine-phosphotransferases (EC 2.7.1.196/205) indicates that microorganisms can not only utilize free sugars but also efficiently degrade polymers such as starch, cellulose, and chitooligosaccharides. This explains part of the reason for the softening of fruit texture after fermentation, and suggests that the fermentation process may release more potential prebiotics (e.g., oligosaccharides). During the fermentation of cocoa pulp-bean mass, 6-phospho-beta-glucosidase was one of the most abundant KEGG genes and was closely associated with the starch and sucrose metabolism pathway as well as the glucose metabolism pathway. This suggests that fermentation may release more potential prebiotics, and that the texture change in cocoa after fermentation is indeed caused by the activity of this type of enzyme [46]. Phosphoserine phosphatase (EC 3.1.3.3) appears in multiple pathways, including glycolysis, the pentose phosphate pathway, and fructose metabolism. The classical function of this enzyme is to participate in serine synthesis. Its co-annotation in several carbohydrate-related pathways may suggest that this enzyme has broad substrate specificity in the fermentation system, or that a functionally similar phosphatase family exists; more likely, it represents the close crosstalk between amino acid metabolism and carbohydrate metabolism. The metabolism of phosphoserine links the glycolytic intermediate 3-phosphoglycerate with the synthesis of glycine and cysteine, serving as a key point of carbon–nitrogen metabolic integration. Its activity may imply a synergistic enhancement of nitrogen assimilation and carbon skeleton supply during fermentation. It regulates the redox balance of stressed plant cells through reactions involved in the glycerate and phosphoserine pathways. These pathways are important processes that connect carbon and nitrogen metabolism and help maintain cellular redox and energy levels under stress conditions [47].

Genomes from Staphylococcus and Xanthomonas encode adenosylhomocysteinase (EC 3.13.2.1), which catalyzes the hydrolysis of S-adenosylhomocysteine into homocysteine and adenosine, a key step in the methyl cycle and methionine regeneration. More importantly, homocysteine is a precursor for the synthesis of cystathionine, cysteine, and ultimately various sulfur-containing flavor compounds (e.g., isothiocyanates). The activity of this enzyme serves as a molecular signal that the fermented product may develop a complex, rich ‘savory’ or ‘fermented’ aroma. In the fermentation system, some aromatic amino acids, including phenylalanine, tyrosine, and tryptophan, can be metabolized to produce volatile flavor compounds such as benzaldehyde, benzyl alcohol, phenylethanol, and phenylacetaldehyde [48]. Phenylalanine can be converted to phenylacetaldehyde via deamination and decarboxylation, and the genome-encoded alcohol dehydrogenase (EC 1.1.1.1) can further reduce it to phenylethanol. Phenylethanol is an aromatic alcohol with a rose-like scent and is an important flavor compound in many fermented foods and beverages (e.g., baijiu, cheese). This is consistent with the hypothesis that fermentative microorganisms may convert plant-derived aromatic amino acids into higher-order flavor compounds. Abundant benzene-type compounds, such as benzaldehyde, phenylethanol, and phenylacetaldehyde, mainly originate from the metabolic transformation via various phenylalanine degradation pathways [49]. Furthermore, phosphoserine phosphatase (EC 3.1.3.3) is again encoded in amino acid metabolism, echoing observations in carbohydrate metabolism. This suggests that the glycolytic intermediate 3-phosphoglycerate may be extensively used for serine synthesis, which is then metabolized to generate glycine and cysteine. This is one of the primary pathways for the flow of carbon skeletons (from sugars) into amino acids. Metabolomics studies have confirmed that 3-phosphoglycerate participates in the synthesis of glycine and cysteine via the serine pathway and further through the one-carbon metabolism network [50,51]. Similarly, it is no coincidence that the genomes of *Lactiplantibacillus* and *Pediococcus* again encode lactate dehydrogenase (EC 1.1.1.27) and acetate kinase (EC 2.7.2.1). This indicates that the lactic acid and acetic acid formed from pyruvate, the end product of glycolysis, can serve as starting points for the synthesis of alanine, valine, leucine, and for entry into the arginine synthesis pathway. This is indicative of the potential metabolic plasticity of the fermentation system in potentially converting carbon sources (sugars) into nitrogenous compounds (amino acids).

Furthermore, genes encoding enzymes related to fatty acid metabolism were also putatively identified in the *Lactiplantibacillus* genome associated with lactobacilli. For example, genes for acetyl-CoA carboxylase (EC 6.4.1.2) and acetyl-CoA carboxytransferase (EC 2.1.3.15) were putatively identified, which are responsible for catalyzing the production of octanoic acid, nonanoic acid, and sorbic acid. Meanwhile, volatile esters can also be synthesized via esterification of corresponding alcohols with fatty acids [52]. It should be noted that while the co-occurrence of specific enzyme-encoding genes and their corresponding flavor compounds is consistent with a functional relationship, the causal linkage remains inferential and requires further experimental validation (e.g., enzyme activity assays, gene knockout studies, or targeted metabolic flux analysis).

## 3. Materials and Methods

### 3.1. Chemicals

Sodium chloride (analytical grade) was obtained from Sinopharm Chemical Reagent Co., Ltd. (Shanghai, China). n-Hexane (chromatographic grade) was purchased from Merck Investment (China) Co., Ltd. (Shanghai, China).

### 3.2. Sample Preparation, Pasteurization, and Fermentation Procedure

*Physalis pubescens* L. fruits were purchased from a local plantation in Muling City, Heilongjiang Province, China (44.9106° N, 130.5258° E). Fresh fruits with uniform size, color, and ripeness were selected, washed thoroughly with sterile distilled water, and manually destemmed. The cleaned fruits were then crushed using a sterile laboratory blender (Joyoung JYL-Y9, Hangzhou, China) at medium speed for 3 min to obtain a homogeneous fruit pulp. No additional water or any exogenous liquid was added during the crushing step to avoid dilution of the natural substrate.

To eliminate background microorganisms and undesirable enzymatic activities, the freshly crushed pulp was immediately subjected to pasteurization in a water bath at 85 °C for 10 min, followed by rapid cooling to room temperature in an ice-water bath. The pasteurization conditions were applied uniformly to both the control (uninoculated, non-fermented group, abbreviated as NF group) and fermentation groups (*Lactiplantibacillus plantarum*-inoculated, fermented for 24 h group, abbreviated as LPF24 group).

A commercial direct-vat-set (DVS) freeze-dried powder of *Lactiplantibacillus plantarum* strain MH-LP-126 (Harbin Meihua Biotechnology Co., Ltd., Harbin, China) was used as the starter culture, with a certified viable count of 10^11^ CFU/g. The DVS powder was added directly to the pasteurized and cooled fruit pulp at a ratio of 0.02% (*w*/*w*) (i.e., 0.2 g of powder per 1000 g of pulp). The mixture was thoroughly homogenized by sterile stirring for 5 min. Fermentation was carried out at 37 °C for 24 h under static conditions. The non-fermented control group (NF) consisted of the pasteurized pulp without inoculation, processed immediately after cooling. The fermentation duration of 24 h was selected based on preliminary time-course experiments monitoring the dynamic changes in pH and total acid (TA) during fermentation.

At each sampling time point (0 h for NF group and 24 h for LPF24 group), three random replicate samples were collected from deep inside each container (approximately 30 cm below the top surface) and thoroughly mixed. A total of six mixed samples were collected (three for NF and three for LPF24). Then, 20 mL of each collected sample was centrifuged at 8000 rpm for 10 min at 4 °C. The supernatant was separated and immediately used for volatile flavor metabolite analysis. The pellet (containing microbial cells and insoluble fruit debris) was collected for metagenomic DNA extraction. Both fractions were immediately frozen in liquid nitrogen and stored at −80 °C until further analysis.

### 3.3. Volatile Flavor Metabolite Analysis

Volatile flavor metabolites were extracted using automated headspace solid-phase microextraction (HS-SPME) and analyzed by gas chromatography–mass spectrometry (GC-MS). Approximately 1 mL of the sample was transferred immediately to a 20 mL headspace vial (Agilent, Palo Alto, CA, USA), containing 1 mL NaCl saturated solution to inhibit any enzyme reaction. The vials were sealed using crimp-top caps with TFE-silicone headspace septa (Agilent). At the time of SPME analysis, each vial was placed in 60 °C for 5 min, then a SPME Arrow (Agilent) of 120 µm DVB/CWR/PDMS was exposed to the headspace of the sample for 15 min at 60 °C.

After sampling, desorption of the VOCs from the SPME Arrow coating was carried out in the injection port of the GC apparatus Model 8890; (Agilent, Palo Alto, CA, USA) at 250 °C for 5 min. The putative identification and internal standard-based relative quantification of VOCs was carried out using an Agilent Model 8890 GC and a 7000D mass spectrometer (Agilent), equipped with a 30 m × 0.25 mm × 0.25 μm DB-5MS (5% phenyl-polymethylsiloxane) capillary column. Helium was used as the carrier gas at a linear velocity of 1.2 mL/min. The injector temperature was kept at 250 °C. The oven temperature was programmed from 40 °C (3.5 min), increasing at 10 °C/min to 100 °C, at 7 °C/min to 180 °C, at 25 °C/min to 280 °C, hold for 5 min. Mass spectra was recorded in electron impact (EI) ionization mode at 70 eV. The quadrupole mass detector, ion source and transfer line temperatures were setat 150, 230 and 280 °C, respectively. The MS was selected ion monitoring (SIM) mode and it was used for the identification and quantification of analytes.

GC-MS analysis was performed in selected ion monitoring (SIM) mode. In SIM mode, the quadrupole mass analyzer sequentially filters ions of specific mass-to-charge ratios (*m*/*z*) by adjusting the electric field parameters, while excluding interfering ions from other mass ranges. This approach significantly enhances detection sensitivity and signal-to-noise ratio, making it particularly suitable for the analysis of trace volatile compounds.

Compound identification and relative quantification were performed using a widely targeted volatilomics method, which integrates multiple identification dimensions. A self-established database was constructed based on multiple species, literature data, partial authentic standards, and retention indices, containing confirmed retention times (RT) and characteristic ions for each compound. Qualitative analysis was performed using Agilent MassHunter software (Qualitative Analysis version 10.0) with deconvolution parameters set as follows: peak width = 20, resolution and sensitivity set to medium, and minimum match factor ≥ 70. Compounds were putatively identified by comparison of their mass spectra with the NIST Mass Spectral Library (NIST 2020). Experimental retention indices (RI) were calculated using a series of n-alkane standards (C7–C40) according. A match within ±30 RI units of published values was required for confirmation. Identifications were further cross-validated against published literature reports.

For detection in SIM mode, each compound was monitored using one quantitative ion and 2–3 qualitative ions, selected based on the self-established database. All ions to be detected were divided into time segments according to their elution order and detected sequentially. A compound was considered positively identified when: (i) the observed retention time matched the reference standard within the established window; and (ii) all selected characteristic ions were present in the background-subtracted mass spectrum.

For internal standard-based relative quantification, the peak area of each compound was normalized against the peak area of the internal standard (3-Hexanone-2,2,4,4-d4). Relative abundances were expressed as the ratio of each compound’s peak area to that of the internal standard. Full structural confirmation would require additional analysis using authentic reference standards. Accordingly, all compound names reported in this study should be interpreted as putative identifications. For each compound, the mean relative abundance (internal standard-normalized peak area ratio) from three technical replicates was calculated and used for subsequent statistical comparisons between groups.

### 3.4. Sample Testing, Library Construction, and Sequencing

DNA concentration was measured using Qubit^®^ dsDNA Assay K it in Qubit^®^ 4.0 Flurometer (Thermofisher Life Technologies, Carlsbad, CA, USA). DNA contents above 0.2 μg were used to construct library. A total amount of 0.2 μg DNA per sample was used as input material for the DNA sample preparations. The input DNA sample was fragmented by the fragmentation reagent to a size of 300 bp, then DNA fragments were end-polished, A-tailed, and ligated with the adaptor for BGI sequencing with further PCR amplificati on. At last, PCR products were purified and libraries were analyzed for size distribution by Qsep400 Bioanalyzer. The constructed libraries (PCR dsDNA) were used with the Circularization Kit to create single-strand circular (ssCir) DNA libraries for subsequent DNB preparation. After DNB preparation, it were directly sequenced on DNBSEQ high-throughput sequencing platforms.

### 3.5. Bioinformatics Analysis

Raw data were quality-controlled using Fastp software V0.19.4 with default parameters to obtain Clean Data for subsequent analyses. To remove potential host contamination, Clean Data were first aligned to a host reference database using Bowtie2, and reads of probable host origin were filtered out [53,54]. The standard alignment parameters for Bowtie2 were set as—sensitive-I 200-X 400, and these parameters were used for all Bowtie2 alignments unless otherwise specified.

After quality control, single-sample assembly was performed with MEGAHIT using the following parameters: k-min 35 k-max 95 k-step 20 min-contig-len 500. Subsequently, the same Bowtie2 parameters were applied to align each sample’s Clean Data to its corresponding assembled contigs, and the unutilised paired-end (PE) reads were collected [55]; unutilized reads of each sample were put together for mixed assembly [53,56], and the assembly parameters were the same as those of single sample.

Based on contigs (≥500 bp) of each sample and mixed assembly, MetaGeneMark version 3.25 [57,58] was used to predict ORF (Open Reading Frame), and the default parameters were used. Based on the predicted results, the predicted genes with length less than 100 nt were filtered out [55,59]. Combine the ORF prediction results of all samples and mixed assembly, and use CD-HIT V4.8.1 [60,61] software to remove the redundancy, so as to obtain the nonredundant initial gene catalog (here, operationally, the nucleic acid sequence encoded by non-redundant continuous genes was called genes). By default, identity 95% and Coverage 90% were used for clustering [57,62], and the longest sequence is selected as the representative sequence. Parameters: -c 0.95, -g 0, -AS 0.9, -g 1, -d 0; Bowtie2 was used to compare Clean Data of each sample to the initial gene catalog, and the number of reads that gene was compared in each sample was calculated [51,52], the same Bowtie2 parameters were used with the additional --end-to-end option. Genes supporting reads ≤ 2 [63] in each sample were filtered out to obtain gene catalog (Unigenes) for subsequent analysis. Based on the number of reads and gene length compared, the abundance information of each gene in each sample was calculated, as shown in the following formula. R is the number of reads compared to the gene, and L is the length of the gene [64].

$$G_{k}=\frac{r_{k}}{L_{k}}\cdot \frac{1}{\sum_{i=1}^{n}\frac{r_{i}}{L_{i}}}$$

### 3.6. Physicochemical Analysis

The pH, color difference (ΔE), and total acid (TA) of the *P. pubescens* samples (NF and LPF24 groups) were determined according to standard analytical procedures. The pH of the samples was measured using a pH meter (FE28, Mettler Toledo, Greifensee, Switzerland). The color parameters (L, a, b) of the samples were determined using a color difference meter (CR-400, Konica Minolta, Tokyo, Japan). The total acid content was determined according to to the method of Kondratenko et al. [45] and titrated with 0.1 M NaOH solution.

### 3.7. Statistical Analyses

All statistical analyses were performed with three biological replicates per group (n = 3), and each sample was analyzed in three technical replicates. Results are expressed as mean ± standard deviation (SD). Principal component analysis (PCA): Unsupervised PCA of the volatile metabolome was performed using the prcomp function from the base package in R software (version 4.1.2; R Foundation for Statistical Computing, Vienna, Austria). Prior to PCA, the data were subjected to unit variance (UV) scaling to normalize the variance of each variable. Orthogonal partial least squares discriminant analysis (OPLS-DA): Supervised OPLS-DA was performed using the MetaboAnalystR package (version 1.0.1) in R software (version 4.1.2). Spearman’s rank correlation analysis was used to calculate the correlations between microbial abundances and volatile flavor metabolite concentrations. Spearman correlation coefficients were calculated using the cor function from the stats package in R software (version 4.3.2). The Spearman correlation coefficient ranges from –1 to 1, with positive values indicating a positive correlation, negative values indicating a negative correlation, and a larger absolute value representing a stronger correlation. An absolute value of 1 indicates perfect correlation. The correlation analysis was performed using the cor function in R software, and the significance test for the correlation was calculated using the corPvalueStudent function in the WGCNA package of R software. The criteria for a significant correlation between differential microorganisms and metabolites were |r| ≥ 0.8 and *p*-value < 0.05 from the significance test of the correlation coefficient.

## 4. Conclusions

This study systematically compared the volatile flavor compounds and microbial community composition of *Physalis pubescens* L. before and after 24 h of fermentation with *Lactiplantibacillus plantarum*. Our findings indicate that *L. plantarum* fermentation is associated with fundamental changes in both the metabolic landscape and the microbial ecology of *P. pubescens*, which may contribute to a transformative enhancement of its flavor profile and safety characteristics.

From a metabolic perspective, fermentation significantly upregulated 501 volatile metabolites, with terpenoids (e.g., D-limonene, geraniol, D-carvone), esters (e.g., β-phenylethyl acetate), phenols, and alcohols (e.g., phenylethyl alcohol) being the most prominently enhanced classes. These compounds collectively impart citrus, floral, fruity, and rose-like aromas to the fermented product, substantially enriching its sensory complexity. The accumulation of these flavor-active compounds is mechanistically linked to the activation of key metabolic pathways including glycolysis, amino acid metabolism, and fatty acid metabolism as revealed by functional gene annotation.

From an ecological perspective, *L. plantarum* rapidly established mono-dominant colonization (from <0.05% to ~72% relative abundance) through a combination of rapid acid production (pH decrease from 4.37 to 3.63), niche occupation, and competitive exclusion of background microbiota. The effective suppression of potential spoilage organisms such as *Escherichia coli* suggests a potential safety-enhancing effect of this fermentation process, which is particularly valuable for extending the shelf life of perishable fruits.

Mechanistically, the close crosstalk between carbohydrate and amino acid metabolism, mediated by key enzymes such as phosphoserine phosphatase (EC 3.1.3.3), is consistent with the hypothesis that *L. plantarum* may efficiently channels carbon skeletons from sugars into amino acid biosynthesis, supporting both rapid growth and the production of flavor precursors. The identification of enzyme genes predominantly assigned to *L. plantarum* including L-lactate dehydrogenase (EC 1.1.1.27), pyruvate oxidase (EC 1.2.3.3), and acetyl-CoA carboxylase (EC 6.4.1.2) provides a molecular basis for understanding the generation of organic acids, ethanol, and ester flavor compounds.

The strong positive correlations between *Lactobacillaceae* genera and key flavor metabolites (terpenoids, phenols, alcohols, and aldehydes) further support the central role of *L. plantarum* and its associated microbiota in flavor formation. Together, these findings establish a comprehensive mechanistic framework for understanding how LAB fermentation transforms the chemical and microbial properties of *P. pubescens*.

While this study focused on a single fermentation time point (24 h) and one *L. plantarum* strain, future research should explore the dynamic evolution of flavor compounds and microbial communities across multiple time points to capture the temporal trajectory of flavor development. Additionally, evaluating the probiotic functionality, prebiotic potential, and sensory acceptance of the fermented product will be essential for translating these findings into industrial applications. Overall, this study provides a robust theoretical and experimental foundation for developing stable, controllable, and high-quality LAB-fermented fruit and vegetable products.

## Figures and Tables

**Table 1:** Major genes and enzymes related to metabolic pathways involved in flavor formation in the genome of inoculated fermented *Physalis pubescens* L.

Enzyme Name	EC Number	Gene	Annotated Microbial Genus
L-lactate dehydrogenase	1.1.1.27	LDH, ldh	*Pantoea*, *Kosakonia*, *Cronobacter*, *Lactiplantibacillus*, *Pediococcus*
alcohol dehydrogenase	1.1.1.1	adhP, E1.1.1.1, adh	*Xanthomonas*, *Kosakonia*, *Pantoea*, *Enterobacter*, *Lactiplantibacillus*, *Pediococcus*
pyruvate oxidase	1.2.3.3	spxB, poxL,	*Lactiplantibacillus*, *Pediococcus*
acetate kinase	2.7.2.1	ackA	*Lactiplantibacillus*, *Pediococcus*
acetyl-CoA carboxylase	6.4.1.2	ACACA, accA, accC, accD	*Xanthomonas*, *Enterobacter*, *Pantoea*, *Kosakonia*, *Blastococcus*, *Lactiplantibacillus*, *Pediococcus*
6-phospho-beta-glucosidase	3.2.1.86	E3.2.1.86B, bglA	*Pediococcus*, *Kosakonia*, *Enterobacter*, *Pantoea*
oligo-1,6-glucosidase	3.2.1.10	IMA, malL	*Lactiplantibacillus*
protein-Npi-phosphohistidine- phosphotransferase196/205	2.7.1.196/205	celC, chbA	*Lactiplantibacillus*, *Pediococcus*, *Lactiplantibacillus*, *Pediococcus*
phosphoserine phosphatase	3.1.3.3	serB, PSPH, rsbX, rsbU_P, psp	*Enterobacter*, *Xanthomonas*, *Kosakonia*, *Pantoea*, *Lactiplantibacillus*, *Pediococcus*
adenosylhomocysteinase	3.13.2.1	AHCY, ahcY	*Kribbella*, *Staphylococcus*, *Xanthomonas*
methylglyoxal reductase (NADPH)	1.1.1.283	yvgN, GRE2	*Klebsiella*, *Xanthomonas*, *Lactiplantibacillus*, *Pediococcus*
acetyl-CoA carboxytransferase	2.1.3.15	ACACA, accA, accD	*Pantoea*, *Xanthomonas*, *Enterobacter*, *Kosakonia*, *Blastococcus*, *Lactiplantibacillus*, *Pediococcus*

## Data Availability

The original contributions presented in the study are included in the further inquiries can be directed to the corresponding authors.
